# Comparison and Modeling of Different Drying Technologies for *Zanthoxylum bungeanum* Maxim.: Changes in Drying Kinetics, Color, Dehiscence Rate, Volatile Oil Content and Amide Content

**DOI:** 10.3390/foods15040734

**Published:** 2026-02-16

**Authors:** Jian-Wu Dai, Qi Zeng, Ying-Qing Du, Yao-Wen Liu, Hong-Wei Xiao, Wen Qin, Ying-Lu Li

**Affiliations:** 1 College of Mechanical and Electrical Engineering, Sichuan Agricultural University, Yaan 625014, China; wangkun416@126.com (Q.Z.); yangwenhua314@126.com (Y.-Q.D.); 2 College of Food Science, Sichuan Agricultural University, Yaan 625014, China; du15883144562@126.com (Y.-W.L.); wus0923@126.com (W.Q.); zq18111300450@126.com (Y.-L.L.); 3 College of Engineering, China Agricultural University, Beijing 100086, China; lh15828452014@126.com

**Keywords:** *Zanthoxylum bungeanum* Maxim., drying kinetics, numerical modeling, quality

## Abstract

This study systematically evaluated the drying kinetics of *Zanthoxylum bungeanum* Maxim. during microwave vacuum drying (MVD), pulsation vacuum drying (PVD) and hot-air drying (HAD) at different temperatures and analyzed the heating mechanism differences in the three technologies via numerical simulation. Drying kinetics indicated that MVD was the most efficient technique owing to its volumetric dielectric heating, whereas the PVD efficiency depended heavily on precise cyclic parameter control. As verified by simulations, a more uniform temperature field was formed in MVD, while PVD achieved focused core heating via infrared radiation. Quality analysis revealed that the dehiscence rate increased significantly with the temperature, and both MVD and PVD demonstrated superior color retention over HAD; however, MVD was the most effective for preserving volatile oils, while PVD excelled in amide preservation. It should be noted that the specific component retention advantages of PVD were balanced by its strict parameter requirements, which limits its potential for large-scale application. Comprehensive evaluation confirmed MVD’s superiority in *Z. bungeanum* drying, effectively retaining thermosensitive components under a vacuum pressure of −90 kPa at 60 °C.

## 1. Introduction

*Zanthoxylum bungeanum* Maxim. (*Z. bungeanum*), a famous spice recognized by its distinctive aroma and special pungency, is rich in volatile oils, amides, alkaloids, and phenolic compounds and is widely distributed in most parts of China [1]. The aromatic components of *Z. bungeanum* constitute valuable sources of essential oils and are highly valued in commercial applications, including food flavoring, traditional medicine and pharmaceutical formulations [2]. Products derived from the processing of *Z. bungeanum* exhibit a range of pharmacological properties, including analgesic, insecticidal, antibacterial, vasodilatory and antihypertensive effects, as documented in several pharmacological studies [3]. Fresh *Z. bungeanum* fruits are prone to rapid enzymatic browning and microbial decomposition under ambient conditions, resulting in postharvest handling challenges and quality deterioration issues [4]. As an important postharvest processing treatment for *Z. bungeanum*, drying enables moisture reduction to inhibit microbial proliferation, extend storage life, ensure quality consistency and facilitate downstream processing [5].

Hot-air drying (HAD) currently dominates the industrial-scale dehydration of *Z. bungeanum* products due to its low operation cost, scalability, diversified form of energy utilization, etc. [6]. However, hot-air drying inevitably causes undesirable quality changes, such as Maillard browning, lipid oxidation and degradation of nutrients and flavor compounds [7,8]. In comparison, vacuum-based drying techniques offer distinct advantages for processing heat-sensitive botanical materials, such as *Z. bungeanum*. Pulsed vacuum drying (PVD) promotes moisture migration by generating micro-channels through cyclic pressure variations, while the concomitant oxygen-limited environment also inhibits enzymatic reactions [9]. Similarly, microwave vacuum drying (MVD) synergistically combines microwave irradiation with a vacuum environment to enable rapid, volumetric heating with minimal thermal degradation [10,11]. Both techniques have been successfully applied in the dehydration of various agricultural products, covering cereal crops [12,13,14], fruits [4,15,16,17] and medicinal herbs [18]. Nonetheless, the conductive heating under static vacuum conditions often results in non-uniform temperature distribution and inefficient moisture removal [19]. These limitations underscore the need for further research aimed at enhancing heat and mass transfer mechanisms and optimizing apparatus processing parameters specifically for *Z. bungeanum* samples.

The temporal evolution of the temperature and moisture profiles during dehydration critically affects the final product quality [20]. Conventional drying studies, primarily assessed through gravimetric methods, are insufficient to characterize the synergistic heat and mass transfer dynamics. They cannot reveal the real-time evolution of the moisture distribution and associated heat transfer mechanisms, which are critical for a fundamental understanding. Computational modeling, particularly multiphysics-coupled approaches, provides a powerful tool to visualize and predict these internal field distributions, thereby supporting mechanistic interpretation and process optimization in *Z. bungeanum* drying [21,22,23]. However, modeling vacuum–thermal processes is challenging due to multiphysics couplings, with existing studies often emphasizing heat transfer while neglecting the coupled moisture transport kinetics [24]. Moreover, direct comparative studies and validated model applications specifically for *Z. bungeanum* dehydration are particularly scarce. Quantitatively comparing the differential mechanistic effects of various drying methods on material internal state evolution poses a significant challenge, hence impeding the establishment of precise, theory-driven guidelines for process optimization. Therefore, this work investigates the synergistic moisture–temperature patterns in *Z. bungeanum* drying through a comparative analysis of various drying techniques, revealing the distinct heat and mass transfer mechanisms and ultimately underpinning strategies for final product quality enhancement.

Therefore, this study aims to: (1) implement a comparative analysis of the HAD, PVD and MVD techniques on the drying kinetics and variations in the quality indicators; (2) establish multivariate parametric evaluation of drying-induced physicochemical quality degradation (colorimetry, volatile retention, structural integrity) based on method-specific performance benchmarks; (3) apply a multiphysics-coupled computational model for simulating the moisture–temperature distribution dynamics in *Z. bungeanum* pericarp tissues.

## 2. Materials and Methods

### 2.1. Samples

The experimental *Z. bungeanum* specimens were sourced from Luzhou, Sichuan Province. Prior to arranged tests, fresh samples were manually cleaned to remove particulates, hermetically sealed in polyethylene pouches and equilibrated in a climate-controlled refrigerator at 80% relative humidity (RH) and 4 ± 1 °C so as to ensure physical-property consistency. The initial moisture content (2.03 ± 0.095 kg/kg in dry basis) was quantified through gravimetric analysis using an oven-drying method at 105 °C for 24 h following AOAC standards [25].

### 2.2. Experimental Apparatus and Arrangements

**Figure 1:**
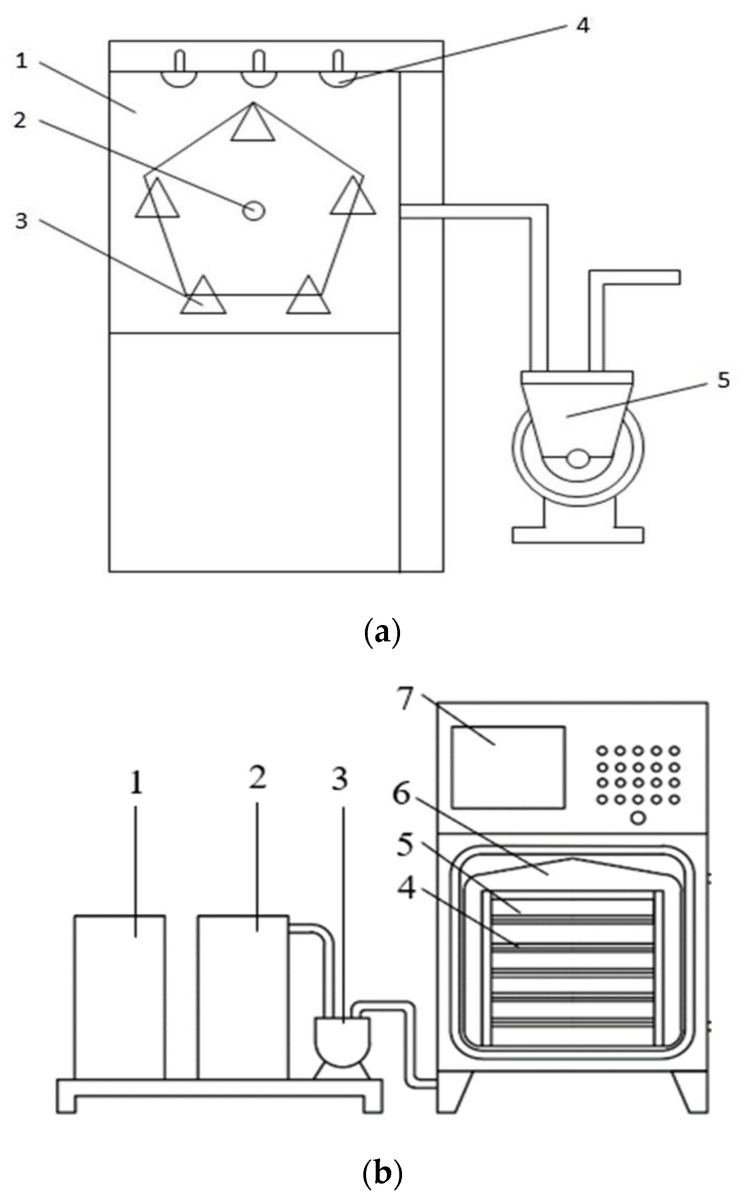
Schematic diagrams of MVD (**a**) and PVD (**b**) drying apparatuses. (**a**) 1. Drying chamber; 2. rotating spindle; 3. drying tray; 4. microwave transmitter; 5. vacuum pump; (**b**) 1. condensing tank; 2. circulating water tank; 3. water-ring vacuum pump; 4. material rack; 5. carbon-fiber infrared heating plate; 6. drying box; 7. control panel.

The MVD system (Figure 1) comprised a stainless-steel process chamber integrated with a 2.45 GHz microwave cavity and vacuum subsystem. Five polypropylene (PP) drying trays were evenly arranged around the central shaft and subjected to horizontal rotation at 5 r/min via the rotary drive device. All samples were arranged in a single, even layer on the trays to facilitate consistent exposure. For MVD operation, the chamber couples hermetically to a refrigerated condensation unit (RCU-300, Leybold GmbH, Deutschland, Germany) and a water-ring vacuum pump (2BV4-2060, Nan-Guang, Zibo, China) with an ultimate pressure of 10 kPa. Tray rotation cyclically modulated the material-to-cavity distance, thereby enhancing heating uniformity. The chamber pressure was monitored by a vacuum transducer (MKS 925B, Andover, MA, USA) at 1 Hz and correspondingly adjusted by the vacuum fine-tuning valve; meanwhile, the material surface temperature was measured through a top-mounted infrared sensor (OS550, Omega Engineering Inc., Storrs, CO, USA) with a measurement accuracy of ±0.5 °C.

The PVD apparatus implemented pulsed pressure cycling (vacuum–atmospheric transitions) through the vacuum pump (2BV, HaoXin Co., Ltd., Shanghai, China) and solenoid-controlled air admittance valves. Based on the preliminary experiment conclusions [11], alternating cycles of a 15 min vacuum phase (10 kPa) followed by a 5 min atmospheric pressure phase were regulated by PID-controlled actuators (Meacon, Hangzhou, China). Far-infrared radiation (FIR-600, Nanjing, China) was selected as the heating source due to its low energy consumption, uniform temperature distribution and rapid heating efficiency [10]. Five infrared carbon heating panels were installed vertically in the PVD chamber with a fixed inter-panel distance of 60 mm. To ensure uniform exposure, the samples were arranged in a single layer on 304 stainless-steel mesh trays and positioned centrally to maintain an equal distance to the infrared panels, thereby achieving symmetrical radiation. Hot-air drying (HAD) was performed as the control group by using a precision-convection oven (FD112, IRM Technology GmbH, Dunwoody, Germany), and the airflow velocity in the HAD was fixed at 1.5 m/s.

The effects of different drying temperatures (40, 50, 60, and 70 °C) with a constant-ambient-humidity condition of 9.8 g/kg on the *Z. bungeanum* quality indicators were investigated in this work. The initial mass for each trial was maintained at 200 ± 5 g with samples arranged in a single layer. The sample weight was measured intermittently until the dry-basis moisture content declined to the safe level of 0.11 kg/kg [6]. For MVD, the optimal power density (microwave power/sample weight) for *Z. bungeanum* was set as 8 W/g according to the preliminary test results [26]. All drying experiments were conducted in triplicate, and the mean values were used for subsequent analysis.

### 2.3. Quality Indicator Evaluation

#### 2.3.1. Drying Characteristic Curve

The moisture ratio (*MR*) was calculated through the following Equation (1) [4]:

(1) $$MR=\frac{M_{t}}{M_{0}}$$

where *M*_0_ and *M_t_* are the initial moisture content and moisture content at time t (h) of *Z. bungeanum*, respectively, both on a dry basis (kg·kg^−1^).

To explicitly quantify the moisture gradient within *Z. bungeanum* during the simulation process, the differences between the volume-averaged moisture ratios in the core region and at the surface boundary were extracted and calculated using COMSOL’s average operator (a_ve_). The defining expression is as follows [27]:

(2) $$\Delta MR=MR_{core}-MR_{surface}$$

where Δ*MR* is the core–surface moisture ratio difference; *MR_core_* represents the average moisture ratio in the central part of the *Z. bungeanum* sample; and *MR_surface_* represents the average moisture ratio at the surface of the *Z. bungeanum* sample.

The drying rate (*DR*) was determined from Equation (3) according to Han et al. [28]:

(3) $$DR=\frac{M_{t_{1}}-M_{t_{2}}}{t_{2}-t_{1}}$$

where *M_t_*_1_ and *M_t_*_2_ represent the moisture content (dry basis, kg·kg^−1^) at time *t*_1_ and *t*_2_ (h), respectively; *t*_1_ and *t*_2_ are the corresponding drying times (h), respectively.

#### 2.3.2. Activation Energy

To quantify the energy barrier associated with moisture removal under different drying methods, assessments of the apparent activation energy (*Ea*) were performed and the *Ea* calculated based on the kinetic modeling of the moisture ratio (MR) data, which were fitted to the thin-layer Page model (Equation (4)) [3]:

(4) $$MR=\exp(-k\cdot t^{n})$$

where *k* is the drying rate constant (min^−1^); *t* is the drying time (min); and *n* is the dimensionless Page exponent. Nonlinear regression was used to obtain the best-fit *k* value for each temperature.

Subsequently, the temperature dependence of the rate constant (*k*) was described by the Arrhenius equation, Equation (5), from which the apparent activation energy (*Ea*) was determined as follows [29]:

(5) $$k=k_{0}\exp(-\frac{Ea}{R\cdot T})$$

where *k_0_* is the pre-exponential factor (min^−1^); *Ea* is the apparent activation energy (J·mol^−1^); *R* is the universal gas constant (8.314 J·mol^−1^·K^−1^); and *T* is the absolute drying temperature (*K*). The *Ea* value for each drying method was obtained from the slope of the linear plot of ln (k) versus 1/*T*.

#### 2.3.3. Color

The chromatic aberration (Δ*E*) was treated as a quantitative metric for chromatic deviation between processed and fresh samples, where lower Δ*E* values indicated superior color fidelity to the reference material. A spectrophotometric colorimeter (CR-400, Konica Minolta, Tokyo, Japan) was employed to measure the CIELAB coordinates (L^*^, a^*^, b^*^) during dehydration. Measurements were performed at different locations in quintuplicate for each sample group. After removal of statistical outliers, the mean value was used for the subsequent calculation of Δ*E* via Equation (6) [30]:

(6) $$\Delta E=\sqrt{{\left(L^{*}-L_{0}\right)}^{2}+{\left(a^{*}-a_{0}\right)}^{2}+{\left(b^{*}-b_{0}\right)}^{2}}$$

where *L*_0_, *a*_0_ and *b*_0_ are the brightness, redness/greenness and blueness/yellowness values of fresh *Z. bungeanum* samples, respectively; L^*^, a^*^ and b^*^ are the brightness, redness/greenness and blueness/yellowness values of *Z. bungeanum* samples after drying treatment, respectively.

#### 2.3.4. Dehiscence Rate

Post-dehydration *Z. bungeanum* specimens were partitioned into triplicate aliquots for morphological analysis. Seed separation was performed using a standardized 4 mm aperture sieve (ASTM E11-20). This facilitated quantification of the dehiscence index through the dichotomous classification of dehiscent and non-dehiscent pericarps. The dehiscence rate was calculated as follows [31]:

(7) $$\mu \;=\;(N_{0}/N_{t})\;\times \;100\%$$

where *N*_0_ represents the dehiscent units, and *N_t_* means the total number of counted specimens. Triplicate measurements per treatment group underwent Chauvenet’s criterion outlier rejection prior to arithmetic mean calculation with ±1.5% SD.

#### 2.3.5. Volatile Oil Content

The volatile oil content in *Z. bungeanum* was determined followed the Quality Classification Standard (LY/T 1652-2005) [32]. Dried specimens underwent mechanical comminution followed by 20-mesh sieve fractionation. An amount of 10.0 g of homogenized powder was refluxed with 250 mL distilled water at 98 ± 2 °C for 4.5 h using a programmable heating mantle until oil-phase stabilization occurred in the graduated collector. The mixture was then cooled and allowed to stand for 1 h to measure the volatile oil content.

#### 2.3.6. Amide Content

The amide content was quantified according to GH/T 1290 (ACFSMC, Beijing, China) with modifications. An amount of 1.0 g of *Z. bungeanum* powder was homogenized with 50 mL HPLC-grade methanol in a flask using vortex mixing (30 s, 2000 rpm). Sequential extraction involved 40 kHz ultrasonication for 30 min followed by 120 min phase equilibration. An amount of 10 mL of clarified supernatant was removed from the extracted solution and diluted to Beer–Lambert law compliance with methanol. Absorbance at λ = 270 nm was measured in triplicate using a dual-beam UV spectrophotometer (Shimadzu UV-2600i, Tokyo, Japan) with 10 mm Suprasil quartz cells, employing methanol as the spectral blank. The amide content of *Z. bungeanum* substances (*X*) was calculated according to the following equation [1]:

(8) $$X=\frac{AKV}{mE}$$

where *A* is the absorbance of the diluted amide substance extract at 270 nm; *V* is the volume of the mixed liquid after ultrasonic extraction (mL); *K* is the supernatant dilution factor; m is the mass of the *Z. bungeanum* powder (g); and *E* is the molar absorptivity (410 nm, L·g^−1^·cm^−1^) for target amides.

#### 2.3.7. Comprehensive Score

The multivariate quality indicators of *Z. bungeanum* exhibited dimensional heterogeneity, requiring data normalization (Equation (9)) to resolve the scale heterogeneity and establish consistent metric scales for comparative analysis [3]:

(9) $$d_{i}=\frac{X_{max}-X_{i}}{X_{max}-X_{min}}$$

where *X_i_* is the actual value of each quality indicator; *X_max_* represents the best value of each quality indicator; *X_min_* means the worst value of each quality indicator.

The weighting coefficient (*W_j_*) represented the relative contribution of individual quality indices to the composite score (*F*), where elevated *W_j_* values indicate enhanced index significance and proportional influence on *F*. These coefficients were derived through the entropy-weighting method (Equation (10)) [12], which objectively assigned weights based on information entropy differentials across normalized datasets:

(10) $$W_{j}=\frac{1-E_{j}}{n^{\prime \prime }-\sum_{j=1}^{n}E_{j}}$$

where *E_j_* is the information entropy. $E_{j}=-{\left(lnm^{\prime }\right)}^{-1}\cdot \sum_{i=1}^{m}p_{ij}lnp_{ij}$. The weighting coefficients (*W_j_*) corresponding to the color difference (Δ*E*), dehiscence rate, volatile oil content and amide content in this paper were defined as 0.21, 0.18, 0.22 and 0.39, respectively.

The composite quality index (*F*) ranges from 1 to 10 on a normalized scale, where elevated values demonstrate positive correlations with superior quality attributes. By integrating Equations (9) and (10), the *F*-index is computationally derived through Equation (11) [31]:

(11) $$F=\sum d_{i}W_{j}$$

where *d_i_* represents the normalized values of the quality indicators of *Z. bungeanum*; *W_j_* is the weight coefficient corresponding to each quality evaluation indicator.

#### 2.3.8. Statistical Analysis

Data analysis and model fitting were performed using SPSS 27.0 (SPSS Inc., Chicago, IL, USA). The significance of differences was assessed by ANOVA with Duncan’s post-test at the 95% confidence interval level (*p* ≤ 0.05).

### 2.4. Model Development

#### 2.4.1. Geometric Model

**Figure 2:**
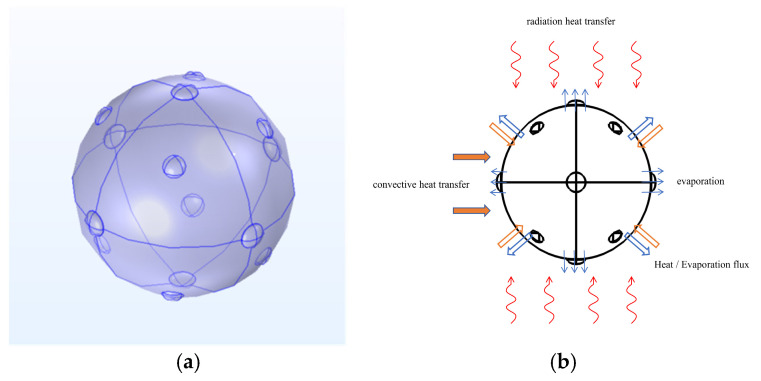
Geometric model (**a**) and heat transfer mechanisms (**b**) of *Z. bungeanum*.

A geometrically accurate model (Figure 2a) with tetrahedral elements was developed using COMSOL Multiphysics v6.2 (COMSOL Inc., Burlington, MA, USA) to characterize the heat and mass transfer in *Z. bungeanum* during the HAD, MVD and PVD processes. The generalized schematic of the coupled heat–mass transport in *Z. bungeanum* during dehydration is presented in Figure 2b. Mesh independence was confirmed via successive refinement, resulting in a final grid with 492,637 degrees of freedom and a maximum element size of 0.0149 m.

#### 2.4.2. Assumptions

The heat and mass transfer models of *Zanthoxylum bungeanum* during MVD, PVD, and HAD were developed and simulated using COMSOL Multiphysics software (version 6.2). The following simplifying assumptions were incorporated into the models [20,22]:

(1) *Zanthoxylum bungeanum* was assumed to be a rigid, homogeneous, and isotropic three-phase (solid, liquid and gas) porous medium. To reduce the computational complexity and focus on the primary transport mechanisms, geometric changes and dynamic porosity due to shrinkage were not explicitly modeled in this study.

(2) Local thermodynamic equilibrium was assumed among the liquid, gas and solid phases.

(3) To represent the net energy input at a macroscale, the absorbed microwave and infrared radiation were simplified to uniform volumetric heating, with the source terms calibrated against experimental drying kinetics for accuracy.

(4) Heat loss through the insulated chamber walls was assumed negligible, confining the model to focus on dominant internal and surface transfer processes, where boundary conditions for convection and radiation with the controlled environment were applied.

#### 2.4.3. Procedure of Multiphysics Simulations

The multiphysics simulations were implemented in COMSOL Multiphysics v6.2, employing the “Heat Transfer in Solids” and “Moisture Transport in Porous Media” physics interfaces. These interfaces were bidirectionally coupled to incorporate the latent heat of evaporation. The material properties listed in Table 1 were assigned to the entire geometry, representing *Zanthoxylum bungeanum* pericarp as a homogenized and isotropic porous medium. A time-dependent study was configured with a total simulation duration matching the experimental drying period. For the transient solution, the Backward Differentiation Formula (BDF) method was used with adaptive time stepping, configured with a user-defined maximum step of 60 s. The relative and absolute solver tolerances were set to 0.01 and 0.001 to ensure numerical convergence, respectively. The model’s performance was evaluated by comparing the simulated and experimental temporal evolutions of the volume-averaged moisture ratio and surface temperature, with the goodness of fit quantified by the root-mean-square error (RMSE) and coefficient of determination (R^2^).

#### 2.4.4. Heat and Mass Transfer Equations

Considering heat transfer and moisture evaporation, the heat transfer equations based on Fourier’s law used to describe the heat transfer inside *Z. bungeanum* were as below [34]:

(12) $$\rho_{m}C_{p}\frac{\partial T}{\partial t}+\rho_{m}C_{p}\cdot \nabla T=k_{p}\nabla T+Q_{IF}+Q_{M}$$

where *ρ_m_* is the density of the sample, kg/m^3^; *C_p_* is the specific heat capacity, J/(kg·°C); *T* is the temperature, °C; *t* is the drying time, min; *Q_IF_* represents the heat-absorbed energy during the PVD drying process, $Q_{IF}=\frac{\kappa S_{m}l_{i}\exp^{-\left(\frac{L}{\delta }\right)}}{V}$; *Q_M_* is the absorbed microwave heat during the MVD process, $Q_{M}=\frac{1}{2}\omega \varepsilon_{0}\varepsilon^{\prime \prime }{\left|E\right|}^{2}$.

Simultaneously, the concentration (*C_w_*) (kg/m^3^) of water transfer within *Z. bungeanum* was calculated from the mass conservation equation, including the convection, diffusion and reaction terms:

(13) $$\frac{\partial C_{w}}{\partial t}+\nabla \cdot \left(-D_{w}C_{w}\right)=-G_{\mathrm{evap}}$$

where *D_w_* is the diffusion coefficient of the material, m^2^/s; *G_evap_* means the evaporation rate governed by the difference between the internal equilibrium vapor pressure and the ambient vacuum pressure, kg/(m^3^·s). In this drying model, *G_evap_* = *Q_evap_*/*L_v_*, where *Q_evap_* represents the energy input necessary to evaporate the moisture contained within a defined volume of Sichuan pepper.

The water diffusion coefficient (*D_w_*) of *Z. bungeanum* was calculated as follows:

(14) $$D_{w}=r^{2}\times \left(-39.41\ln M_{t}-8.247\right)/T$$

where *M_t_* is the dry-basis moisture content of the material, and *r*^2^ was derived from experimental data as well as model simulations.

#### 2.4.5. Boundary Conditions

The boundary conditions are as follows:

(15) $$-k_{m}\frac{\partial T}{\partial t}=h_{t}(T-T_{a})$$

(16) $$-D_{w}\frac{\partial M}{\partial t}=h_{m}(M-M_{e})$$

where *k_m_* is the thermal conductivity of *Z. bungeanum*, W/(m·°C); *h_t_* is the heat transfer coefficient, W/(m^2^·°C); *T* is the material temperature, °C; *T_a_* is the drying air temperature, °C; *h_m_* is the mass transfer coefficient, m/s; *M_e_* is the equilibrium dry-basis moisture content, g/g.

The boundary conditions and heat source terms were defined to reflect the distinct mechanisms of each drying technique. For all simulations, the convective heat transfer coefficient (*h_t_*) and mass transfer coefficient (*h_m_*) at the sample surface were set to 25 W/(m^2^·°C) and 0.025 m/s, respectively, estimated from empirical correlations for natural/mild forced convection under the experimental conditions. For MVD, a uniform volumetric heat source term (*Q_M_* = 8 W/g) was applied to represent the microwave energy absorption. For PVD, the infrared radiant flux, calculated as *li* from Table 1, was applied as a heat flux boundary condition on the exposed surfaces of the model. Conversely, the HAD process was modeled by applying the convective heating equation (Equations (15) and (16)) at the boundaries.

The moisture variation was coupled with the heat transfer through the porous media moisture transport. The steam/water parameters were sourced from COMSOL’s built-in database and the calculations. The initial conditions included a dry-basis moisture content of *M*_0_ = 19 kg/kg and a temperature of *T*_0_ = 20 °C, with the other parameters specified in Table 1. The MVD and PVD vacuum–atmospheric pulsation cycle was implemented using COMSOL’s Piecewise Function Module, configured to feature smooth transitions with continuous second-order derivatives and a transition zone width of 0.08.

## 3. Results and Discussion

### 3.1. Drying Kinetics

**Figure 3:**
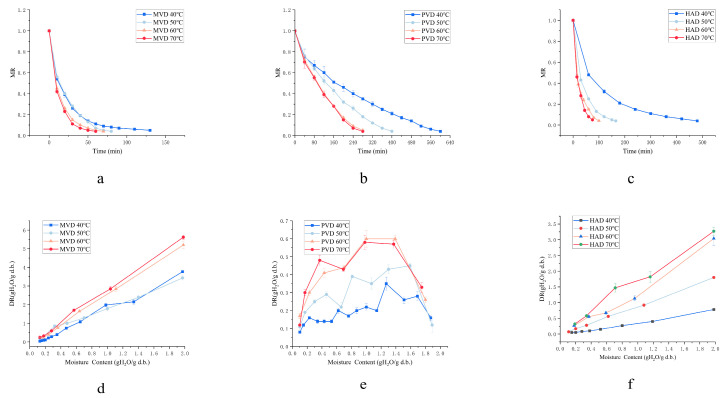
Drying kinetics curves of *Z. bungeanum* under different drying methods. (**a**) moisture ratio curves under MVD; (**b**) moisture ratio curves under PVD; (**c**) moisture ratio curves under HAD; (**d**) drying rate curves under MVD; (**e**) drying rate curves under PVD; (**f**) drying rate curves under HAD.

The drying characteristic curves of *Z. bungeanum* under HAD, MVD and PVD across varied drying temperatures are shown in Figure 3. As expected, elevating the drying temperature from 40 °C to 70 °C significantly increased the drying rate (*p* < 0.05) and shortened the drying time from 150 to 60 min for MVD, from 640 to 260 min for PVD, and from 480 to 75 min for HAD. To enable a quantitative comparison, *E_a_* was determined for each method from the drying rate constants fitted with the Page model. The *E_a_* values were 25.3 ± 1.2, 32.1 ± 1.5, and 38.7 ± 1.8 kJ/mol for MVD, PVD, and HAD, respectively. The lowest *E_a_* for MVD indicated a reduced energy barrier for moisture migration, attributed to its synergistic internal volumetric heating and pressure-driven mass transfer, which collectively explain its superior drying efficiency.

The drying kinetics analysis of *Z. bungeanum* under MVD revealed a temperature-dependent drying duration and high dehydration efficiency compared to HAD, attributable to microwave penetration that enables simultaneous volumetric heating and rapid moisture diffusion [11]. The surface drying kinetics of *Z. bungeanum* followed a declining trajectory throughout MVD (Figure 3d), attributable to the limited microwave-induced thermal generation within the material kernels under fixed microwave power conditions [34].

In contrast, PVD exhibited a transient acceleration in the drying rate at high moisture contents, which was attributed to the internal diffusion rate surpassing the surface evaporation rate, as the cyclic vacuum pulses facilitated more efficient pore diffusion [35,36]. The intermediate *E_a_* of PVD can be attributed to its dual-phase mechanism: the vacuum phases reduced the moisture diffusion barrier, whereas the static infrared heating provided a lower thermal driving force compared to the intense volumetric heating in MVD. This was followed by falling-rate periods dominated by internal moisture migration through seed–husk separation cavities in later stages [37]. Ultimately, progressive moisture reduction led to universally diminished drying rates across all methods toward the end of the drying process.

### 3.2. Heat and Mass Transfer Characteristics

#### 3.2.1. Model Validation

Model validation was conducted by comparing simulated results with experimental data for the three drying methods (MVD, PVD and HAD at 50 °C) in this study. *Z. bungeanum* samples with actual diameters of 5 mm were arranged in drying chambers (400 mm × 270 mm × 20 mm) for method-specific simulations. Real-time thermal monitoring was achieved through integrated temperature sensors, while the evolution of the moisture content was quantified gravimetrically. The drying process was terminated upon reaching a final moisture content of 0.11 kg/kg (dry basis).

The accuracy of the simulation model was quantitatively evaluated using the coefficient of determination (R^2^) and root-mean-square error (RMSE), which served as metrics for the agreement between experimental and predicted values. Higher R^2^ (closer to 1) and lower RMSE values indicate better model performance [33]. The calculated R^2^ and RMSE values for all three drying methods are summarized in Table 2.

**Figure 4:**
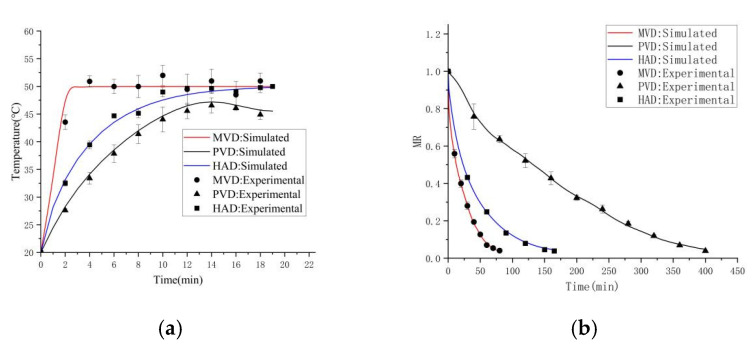
Model validation of MVD, PVD and HAD at 50 °C: (**a**) temperature variation, and (**b**) moisture variation.

Thermal validation (Figure 4a) demonstrated strong concordance between the simulated and experimental surface temperatures of *Z. bungeanum* during drying, with maximum relative deviation below 10%. The quantitative agreement was universally evidenced by high R^2^ values (≥0.91) and low RMSE values (≤2.5 °C) across all drying methods (Table 2). Fresh samples demonstrated rapid initial temperature escalation characteristics, notably with the MVD group showing the most pronounced heating rate due to dielectric polarization. Subsequently, the temperature rise within *Z. bungeanum* kernels plateaued, consistent with the moisture loss-induced thermal equilibration phenomena reported by Alfiya et al. [38] in the spice-drying process. Moisture transfer validation (Figure 4b) indicated high fidelity between the simulated and experimental moisture ratio profiles. This agreement is quantified by the high R^2^ (≥0.93) and low RMSE (≤0.045) values listed in Table 2, verifying the prediction accuracy of the theoretical simulation models. Rapid moisture reduction during the initial drying phase correlated with thermal gradient-driven mass transfer; meanwhile, the subsequent attenuation of the MR value arose from progressive moisture depletion, further degrading the rates of vaporization and overall thermal transfer efficiency [33]. The close agreement between the simulated and experimental data, as supported by the statistical metrics, confirmed the robustness of the model in predicting the coupled heat and mass transfer during *Z. bungeanum* drying.

#### 3.2.2. Heat Transfer Analysis

**Figure 5:**
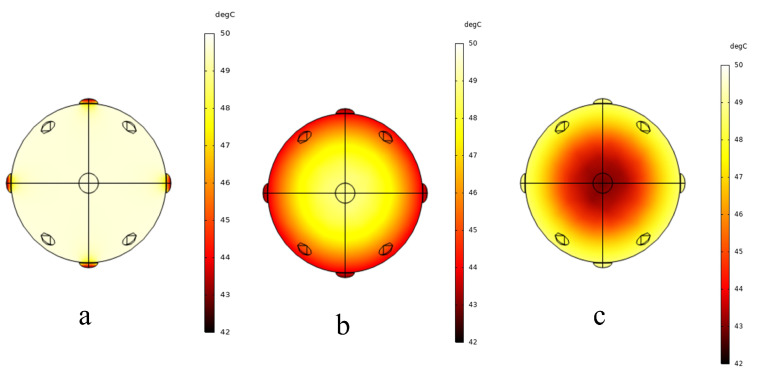
Temperature and electric field distribution inside Z. bungeanum under different drying methods ((**a**) MVD, 20 min; (**b**) PVD, 20 min; (**c**) HAD, 20 min).

Figure 5a–c demonstrate the distinct internal temperature gradients across *Z. bungeanum* after 20 min of drying, with the chromatic intensity correlating to thermal elevation. HAD exhibited a radially decreasing thermal gradient (Figure 5c), with the surface reaching a maximum of 49 °C and the geometric center a minimum of 42 °C, resulting in a 7 °C gradient. The characteristic thermal distribution was attributable to its convective heating mechanism [39]. This convection-dominated regime resulted in sequential conductive lag from surface to core, generating spatiotemporal temperature disparities. Concurrent surface moisture evaporation further intensified the inverse thermal profile through latent heat absorption [40]. Contrary to HAD, PVD produced a reversed thermal gradient from core to surface (Figure 5b), with the core temperature (48 °C) being 5 °C higher than the surface (43 °C). This inverse profile was attributed to the penetrating nature of infrared radiation following Lambert’s law [41]. The infrared radiation penetration accelerated the internal temperature elevation, establishing a thermal field characterized by centralized heating and peripheral cooling through the interaction of radiative heat transfer and surface evaporation, which was corroborated by Shi et al. [32] in the hygrothermal coupling of rice. Meanwhile, the volumetric and internal nature of the dielectric heating in MVD resulted in a remarkably uniform thermal field (Figure 5a), evidenced by a temperature differential of less than 1 °C between the surface and core.

Comparatively, MVD demonstrated a superior heating uniformity over the 20 min dehydration process, as evidenced by its minimal thermal gradient (<1 °C). This stands in sharp contrast to the pronounced gradients in HAD (7 °C) and PVD (5 °C), quantitatively highlighting its critical energy delivery homogeneity. This stabilization arose from rapid dielectric heating achieving target temperatures within minutes, followed by equilibrium-phase drying with minimal thermal fluctuations [22].

#### 3.2.3. Mass Transfer Analysis

**Figure 6:**
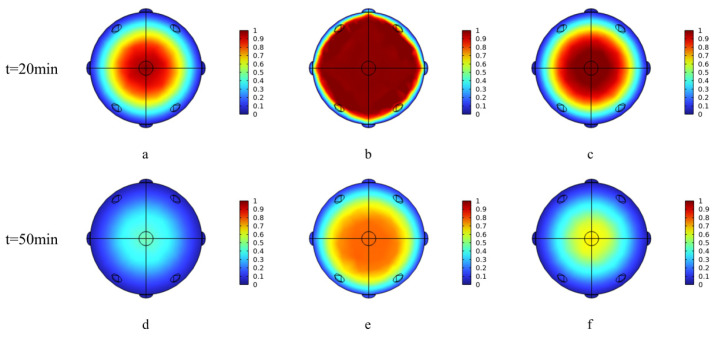
Cross-sectional moisture distribution in *Z. bungeanum* under various drying conditions ((**a**) MVD, 20 min; (**b**) PVD, 20 min; (**c**) HAD, 20 min; (**d**) MVD, 50 min; (**e**) PVD, 50 min; (**f**) HAD, 50 min).

Figure 6 illustrates the internal moisture distribution profiles within *Z. bungeanum* under the three drying methods at the early-drying (20 min) and mid-drying (50 min) stages, with chromatic mapping describing hygrothermal gradients. The moisture redistribution dynamics were quantified by the core-to-surface moisture ratio difference (ΔMR). Pronounced moisture gradients emerged early at 20 min, with ΔMR values of 0.35, 0.41, and 0.38 for MVD, HAD, and PVD, respectively, indicating strong initial driving forces for internal migration; these gradients progressively diminished by 50 min, and the ΔMR values were reduced to 0.18, 0.22, and 0.25, respectively. Notably, MVD achieved the smallest final gradient (0.18), demonstrating its superior efficacy in promoting moisture uniformity and mitigating internal stress during the mid-stage of drying. During the different drying phases (at 20 and 50 min), moisture retention was the highest in PVD, followed by HAD and then MVD, as evidenced by the thermal dynamics presented in Figure 3. Notably, although rapid moisture loss occurred across all methods, the PVD group exhibited a sustained increase in its moisture migration rate at 50 min (Figure 6e), whereas the migration rates of the HAD and MVD groups declined to 51% and 42% of their 20 min values, respectively. The anomalous drying rate elevation in PVD might have originated from its pressure-cycling mechanism [42]. During the initial stage, thermal inertia delayed moisture removal, while subsequent vacuum pulsations enhanced the structural porosity, reducing the internal mass transfer resistance and facilitating vapor pressure gradients between the core and surface that drive persistent dehydration kinetics [43].

### 3.3. Color

Table 3 identifies the color parameters (L^*^, a^*^, b^*^, Δ*E*) as critical quality indicators for *Z. bungeanum* dehydration. Systematic reductions were found in all chromatic values relative to untreated samples, with MVD and PVD exhibiting heightened parametric sensitivity in the L^*^ and b^*^ coordinates compared to the a^*^ stability. This phenomenon can be attributed to the inhibited enzyme functionality and attenuated oxidative chlorophyll decomposition [34] under combined thermal–vacuum exposure.

**Figure 7:**
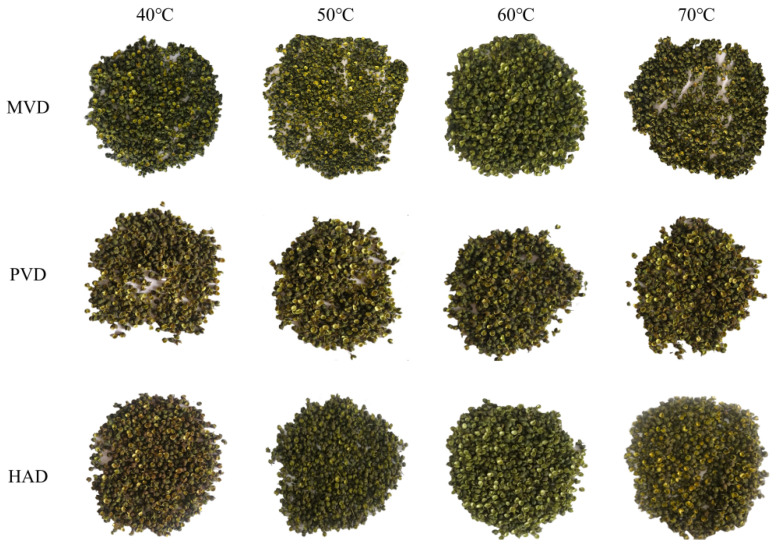
Dried sample drawings of *Z. bungeanum* under MVD, PVD and HAD at different drying temperatures.

As visually documented in Figure 7, the drying temperature exerted a predominant influence on the color attributes in *Z. bungeanum*, resulting in a convex Δ*E* response between 40 °C and 70 °C (Table 3). Comparative Δ*E* analysis confirmed superior color retention in MVD and PVD versus HAD. This synergy slowed chlorophyll degradation by reducing the internal phase transition temperatures and improving the mass transfer kinetics [44]. Specifically, the Δ*E* of the PVD sample dried at 50 °C obtained the minimum value of 2.52. Color difference values initially increased but subsequently declined with rising temperatures in MVD and PVD, whereas HAD conversely demonstrated a persistent increase across these parameters. The observed divergence originated from the dual-phase biochemical kinetics. Prolonged dehydration at 40 °C facilitated enzymatic/non-enzymatic browning through PPO and POD-catalyzed phenolic oxidation [28], while accelerated drying above 60 °C induced Maillard reaction kinetics, manifested by charred-black discoloration [26].

### 3.4. Dehiscence Rate

It can be seen that the dehiscence rate of *Z. bungeanum* employing the MVD, PVD, and HAD methods exhibit significant temperature-dependent elevation characteristics (*p* < 0.05) in Table 3. When the drying temperatures exceeded 60 °C, thermal intensification within defined parameters markedly enhanced the pericarp rupture efficiency. Specifically, the dehiscence rate in the HAD, MVD and PVD groups exhibited notable rises from 97.10% to 99.77%, from 97.87% to 99.31%, and from 91.46% to 98.31%, respectively. This phenomenon may be attributed to the accelerated surface moisture evaporation at elevated temperature induced by rapid pericarp contraction and structural deformation [31]. Concurrently, the internal seed morphology resisted pericarp contraction, generating sufficient compressive stress conducive to fissure formation at *Z. bungeanum* exocarp junctions [42]. When this stress exceeded the mechanical strength of the pericarp at its natural closure point, cracking initiated correspondingly and propagated under continued drying, ultimately facilitating seed detachment. The proposed stress-induced fracture mechanism was consistent with the observed temperature-dependent trends. However, direct validation through measurements of the pericarp mechanical properties (e.g., tensile strength, elastic modulus) remains a subject for further studies to quantitatively substantiate this hypothesis.

### 3.5. Volatile Oil and Amide Content

Table 3 demonstrates a parabolic trend in the volatile oil and amide contents of *Z. bungeanum* under incremental drying temperatures (40–70 °C) across the MVD, PVD and HAD treatments, with minimum concentrations observed at 40 °C. This suggests that moderate temperature elevation (below 60 °C) mitigated phytochemical losses by optimizing the dehydration kinetics, whereas subcritical drying at 40 °C prolonged the dehydration duration and therefore led to extended volatilization periods for labile constituents. Conversely, temperatures exceeding 60 °C accelerated oxidative deterioration through excessive energy input and induced the molecular destabilization of heat-sensitive compounds [30].

**Figure 8:**
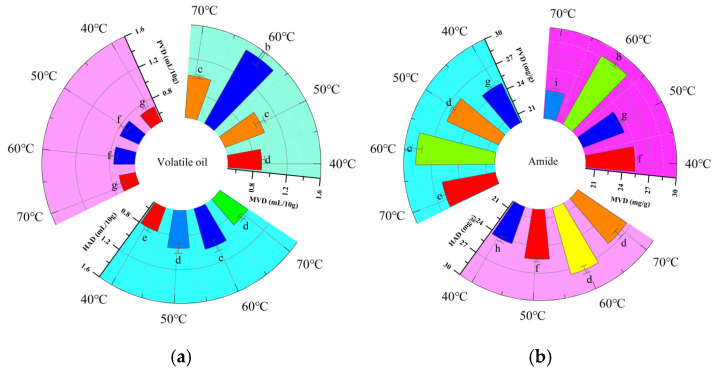
The volatile oil and amide contents of Zanthoxylum bungeanum under different conditions ((**a**) volatile oil; (**b**) amide content). Note: Different lower-case letters in the same figure indicate significant differences (*p* < 0.05).

It can be seen that (Figure 8) MVD significantly enhanced the volatile oil retention versus the other drying methods (*p* < 0.05). The maximum volatile oil content of 1.5 mL/10 g was obtained under a fixed MVD vacuum level of −90 kPa at 60 °C, revealing that the vacuum environment enhanced the phytochemical content by inhibiting oxygen interfacial contact, thereby suppressing oxidative degradation pathways [11]. Meanwhile, the amide content exhibited an oscillatory pattern variation and attained a minimum value of 23.09 mg/g at 70 °C, which was mainly attributed to the decomposition of thermosensitive amides under intensified heating [45]. In contrast, PVD demonstrated no significant differences in the volatile oil content across the various drying temperatures. The maximum amide content of 28.65 mg/g was observed at 60 °C with a vacuum/atmospheric cycle ratio of 15 min: 5 min, which was 1.04 times higher than that in the HAD group. This aligned with the findings of Liu et al. [46], who highlighted the role of the vacuum retention time in PVD as a destabilizing factor for amide stability in *Z. bungeanum*. The concomitant reduction in the boiling point under vacuum conditions also likely contributed to the compromised stability of these thermosensitive compounds.

Comparative analysis using comprehensive evaluation scores (Table 3) revealed that excessive heating temperatures (above 60 °C) resulted in significant degradation across all drying methods, establishing 60 °C as the critical threshold for preserving thermolabile compounds in *Z. bungeanum*. Notably, PVD showed marked advantages in the retention of specific amides, despite a reduction in the volatile oil content, with comprehensive scores (F) ranging from 2.56 to 5.40. In contrast, HAD yielded inferior comprehensive scores (3.18 to 4.29) and induced noticeable chromatic alterations, consistent with conventional thermal degradation kinetics [18]. In addition, MVD significantly enhanced the retention of volatile oils (28.63 mg/g) and amide contents (1.5 mL/10 g) under optimized conditions with a vacuum pressure of −90 kPa at 60 °C, achieving a dehiscence rate of 99.10% and a color difference (Δ*E*) of 3.81, along with the highest comprehensive score (F) of 8.42. These advantages support the recommendation of MVD as a superior processing technique for *Z. bungeanum*, contributing to the improved retention of the bioactive compounds and sensory properties [47].

### 3.6. Schematic Analysis

**Figure 9:**
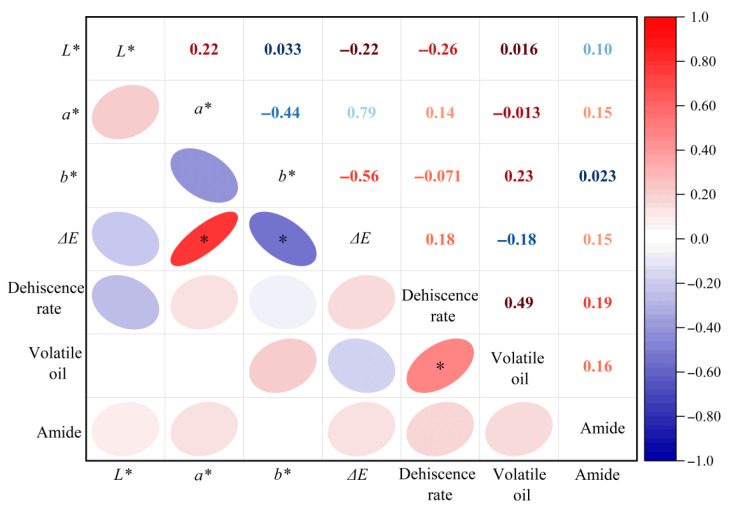
Correlation matrix between dried components and other quality indicators of *Z. bungeanum*. Note: * denotes significant correlation (*p* < 0.05).

Pearson’s correlation analysis was employed to investigate the linear relationships between the color parameters (L^*^, a^*^, b^*^, Δ*E*) and dehiscence rate, volatile oil content and amide content. As illustrated in Figure 9, a^*^ demonstrated a highly significant positive correlation with Δ*E* (r = 0.791, *p* < 0.01), suggesting that alterations in the a^*^ values primarily driven by Maillard reaction and phenolic oxidation during drying served as the key contributor to the accumulation of the total color difference (Δ*E*). A significant negative correlation was observed between b^*^ and Δ*E* (r = −0.560, *p* < 0.05), indicating that higher b^*^ values were associated with lower Δ*E* values. Additionally, the dehiscence rate was significantly and positively correlated with the volatile oil content (r = 0.489, *p* < 0.05), implying a synergistic variation between the two variables. No significant correlations were found between L^*^, a^*^ and Δ*E* or between b^*^ and the dehiscence rate (*p* ≥ 0.05), suggesting an absence of significant linear relationships between these variables.

The above correlations offered preliminary statistical insights; however, their interpretation warrants caution due to the inherent linearity assumption and the potential confounding from the varying drying conditions. Future research needs to be conducted with appropriate multiplicity corrections to substantiate these observed correlations.

## 4. Conclusions

This study systematically investigated the heat and mass transfer mechanisms of *Z. bungeanum* during MVD, PVD and HAD through the implementation of three-dimensional multiphysics models. The drying kinetics demonstrated that MVD achieved the most rapid drying rate owing to its volumetric heating mechanism, whereas PVD exhibited an initially slower heating phase. Computational results exhibited high congruence with experimental data, validating the model fidelity in predicting spatiotemporal temperature/moisture gradients across drying modalities. It can be seen from the numerical simulations that HAD produced a temperature gradient decreasing from the surface to the core, PVD generated focused heating in the internal regions, while MVD facilitated uniform temperature distribution.

Compared to HAD, both MVD and PVD effectively inhibited non-enzymatic browning through synergistic thermal and vacuum effects. MVD was optimal for retaining volatile oils, whereas PVD excelled in preserving heat-sensitive amides with cycling parameters. Comprehensive quality evaluation confirmed the superior performance of MVD in processing *Z. bungeanum*, as evidenced by the highest comprehensive score (F) of 8.42 under optimized conditions of 60 °C and a vacuum pressure of −90 kPa. Statistical analysis revealed the significant positive correlations between the redness value (a^*^) and total color difference (Δ*E*) (*p* < 0.01) and between the dehiscence rate and volatile oil content (*p* < 0.05), while there was a negative correlation between b^*^ and Δ*E* (*p* < 0.05).

Although the findings and correlations offered preliminary insights, the interpretations were context-dependent due to the multiphysical model assumptions and potential confounding from the drying conditions. Further research would be valuable to assess the transferability of the models under broader conditions to fully substantiate the proposed stress-induced fracture hypothesis, evaluate the practical scalability of optimized drying methods, and verify the statistical robustness across variable material and processing parameters, providing better support for agricultural drying practices.

## Figures and Tables

**Table 1:** Operating parameters for simulation of *Z. bungeanum* samples.

Parameter	Symbol	Value	Unit	Reference
Microwave frequency	*f*	2.45	GHz	Parameter of apparatus.
Radiation intensity	*l_i_*	6.54 × 10^3^	W/m^2^	The rated power of the heating panels divided by their effective irradiation area on the sample surface.
Absorption coefficient	*K*	0.9		[33]
Dielectric constant	ɛ′	40		Determined by dielectric constant measuring instrument with similar moisture content and composition at 2.45 GHz.
Dielectric loss	ɛ″	17		
Density	*ρ_m_*	1030	kg/m^3^	Measured using water displacement method for fresh *Z. bungeanum* samples.
Heat transfer coefficient	*k_p_*	0.55	W/(m·°C)	[3]
Specific heat capacity	*C_p_*	1569	J/(kg·°C)	[3]
Liquid water concentration	*C_w_*	707.61	kg/m^3^	Calculated from initial dry-basis moisture content and measured density.
Latent heat of evaporation	*L_v_*	2.32 × 10^6^	J/kg	[22]
Evaporation rate constant	*K_evap_*	1 × 10^−6^		[12]
Constants in Antoine equation	A	7.8087		[34]
Constants in Antoine equation	B	1007.839		[34]
Constants in Antoine equation	C	−166.3583		[34]

**Table 2:** Comparison of simulated and experimental data for *Z. bungeanum* under different drying methods at 50 °C.

Drying Method	Moisture Ratio (MR)		Temperature (°C)	
	R^2^	RMSE	R^2^	RMSE
MVD	0.96	0.032	0.94	1.5
PVD	0.93	0.045	0.91	2.2
HAD	0.95	0.038	0.92	2.5

**Table 3:** Effects of different drying techniques and drying temperatures on quality attributes of *Z. bungeanum*.

Drying Methods		Color				Dehiscence Rate/%	Volatile Oil Content/mL·10/g	Amide Content/mg/g	Comprehensive Score (F)
		*L* ^*^	a^*^	b^*^	Δ*E*				
Fresh sample		31.82 ± 0.27 ^a^	−21.23 ± 0.48 ^h^	19.51 ± 0.39 ^a^	-	-	2.80 ± 0.16 ^a^	55.06 ± 0.42 ^a^	/
MVD	40 °C	25.94 ± 0.26 ^g^	−17.68 ± 0.31 ^c^	16.35 ± 0.34 ^f^	6.73 ± 0.18 ^b^	97.87 ± 0.63 ^h^	0.90 ± 0.14 ^d^	25.39 ± 0.33 ^f^	1.82
	50 °C	28.91 ± 0.26 ^d^	−18.24 ± 0.32 ^d^	17.39 ± 0.17 ^d^	3.75 ± 0.24 ^i^	98.09 ± 0.36 ^g^	1.00 ± 0.06 ^c^	24.84 ± 0.28 ^g^	4.00
	60 °C	29.42 ± 0.35 ^d^	−17.17 ± 0.09 ^b^	18.07 ± 0.21 ^c^	3.81 ± 0.16 ^h^	99.10 ± 0.76 ^c^	1.50 ± 0.09 ^b^	28.63 ± 0.22 ^b^	8.42
	70 °C	28.74 ± 0.30 ^d^	−16.49 ± 0.35 ^a^	16.17 ± 0.09 ^f^	5.59 ± 0.24 ^e^	99.31 ± 0.58 ^b^	1.00 ± 0.02 ^c^	23.09 ± 0.36 ^i^	3.68
PVD	40 °C	30.23 ± 0.22 ^c^	−17.72 ± 0.24 ^c^	16.22 ± 0.02 ^f^	4.40 ± 0.20 ^g^	91.46 ± 0.73 ^j^	0.70 ± 0.04 ^g^	24.83 ± 0.19 ^g^	2.56
	50 °C	30.06 ± 0.06 ^c^	−18.27 ± 0.18 ^d^	18.41 ± 0.37 ^b^	2.52 ± 0.20 ^k^	97.19 ± 0.34 ^i^	0.75 ± 0.5 ^f^	26.28 ± 0.20 ^d^	4.90
	60 °C	28.19 ± 0.10 ^e^	−18.65 ± 0.19 ^e^	16.86 ± 0.09 ^e^	4.37 ± 0.07 ^g^	98.20 ± 0.52 ^f^	0.75 ± 0.04 ^f^	28.65 ± 0.56 ^c^	5.40
	70 °C	29.48 ± 0.10 ^d^	−16.63 ± 0.40 ^a^	15.46 ± 0.37 ^g^	5.70 ± 0.29 ^d^	98.31 ± 0.24 ^e^	0.70 ± 0.5 ^g^	25.94 ± 0.41 ^e^	4.02
HAD	40 °C	28.94 ± 0.21 ^d^	−20.11 ± 0.29 ^f^	17.80 ± 016 ^d^	3.07 ± 0.21 ^j^	97.10 ± 0.26 ^i^	0.80 ± 0.04 ^e^	24.31 ± 0.31 ^h^	3.18
	50 °C	26.38 ± 0.22 ^f^	−19.30 ± 0.15 ^g^	17.48 ± 0.19 ^d^	5.44 ± 0.29 ^f^	98.20 ± 0.59 ^f^	0.95 ± 0.06 ^d^	25.48 ± 0.29 ^f^	3.98
	60 °C	31.35 ± 0.40 ^b^	−16.43 ± 0.35 ^a^	14.99 ± 0.12 ^h^	5.86 ± 0.34 ^c^	98.43 ± 0.78 ^d^	1.00 ± 0.07 ^c^	27.60 ± 0.43 ^d^	4.29
	70 °C	26.58 ± 0.94 ^f^	−16.20 ± 0.40 ^a^	13.58 ± 0.38 ^i^	8.68 ± 1.22 ^a^	99.77 ± 0.16 ^a^	0.90 ± 0.03 ^d^	26.49 ± 0.44 ^d^	3.45

Note: Different lower-case letters in the same column indicate significant differences (*p* < 0.05)

## Data Availability

The data that support the findings of this study are available from the corresponding author upon reasonable request.
